# Impact of Small Groups with Heterogeneous Preference on Behavioral Evolution in Population Evacuation

**DOI:** 10.1371/journal.pone.0121949

**Published:** 2015-03-20

**Authors:** Tao Wang, Keke Huang, Zhen Wang, Xiaoping Zheng

**Affiliations:** 1 Department of Automation, Tsinghua University, Beijing, China; 2 Interdisciplinary Graduate School of Engineering Sciences, Kyushu University, Kasuga-koen, Kasuga-shi, Fukuoka 816-8580, Japan; Tianjin University of Technology, CHINA

## Abstract

Up to now, there have been a great number of mechanisms to explain the individual behavior and population traits, which seem of particular significance in evolutionary biology and social behavior analysis. Among them, small groups and heterogeneity are two useful frameworks to the above issue. However, vast majority of existing works separately consider both scenarios, which is inconsistent with realistic cases in our life. Here we propose the evolutionary games of heterogeneous small groups (namely, different small groups possess different preferences to dilemma) to study the collective behavior in population evacuation. Importantly, players usually face completely different dilemmas inside and outside the small groups. By means of numerous computation simulations, it is unveiled that the ratio of players in one certain small group directly decides the final behavior of the whole population. Moreover, it can also be concluded that heterogeneous degree of preference for different small groups plays a key role in the behavior traits of the system, which may validate some realistic social observations. The proposed framework is thus universally applicable and may shed new light into the solution of social dilemmas.

## Introduction

In evolutionary biology and social science, a challenging problem is to understand the emergence of collective behavior traits and their sustenance under the pressure of a series of competing strategies [1–5]. To resolve the above issue, evolutionary game theory, which provides a useful framework, has been extensively investigated from theoretical and experimental viewpoints over the past decades [6–11]. In particular, the pairwise interaction game, as a simple metaphor of illustrating the social conflict between different behaviors, has attracted great interest [12, 13]. Typical examples include prisoner’s dilemma game [14–16], snowdrift game [14, 17], harmony game [18, 19] and ultimate game [20]. The former three mainly focus on the origin of cooperation, while the remaining latter gives attention to fairness. Under different scenarios, different payoff ranking will lead to various dilemmas. For example, in prisoner’s dilemma game, players are likely to choose the selfish defection behavior if they wish to maximize individual benefit, irrespective of the fact that mutual cooperation could yield a higher collective benefit [9, 12]. On the other hand, in the snowdrift game, it is worth to cooperate, whatever the opponent does, and thus the number of cooperator-defector pairs arises [6, 12].

To explain these observations, several mechanisms that support the evolution of collective behavior have been identified [11, 21–24], such as the tit-for-tat [25] or win-stay-lose-shift [26], spatially structured populations [10, 23, 27–30], punishment and reward [31], reputation [32], voluntary participation [33], heterogeneity [34, 35] and diversity [36–38], the mobility of agents [39, 40], scale of interaction network [41] and co-evolutionary rules [42]. While recently, Nowak attributed all these to five mechanisms: kin selection, direct reciprocity, indirect reciprocity, network reciprocity, and group selection [21], these mechanisms can be somewhat related to the reduction of an opposing player’s anonymity relative to the so-called well-mixed situation. After this seminal survey, more related scenarios have been intensively explored. In a recent research [7], where the partner selection of agents was closely related with individual reputation, it was found that collective cooperation behavior would be guaranteed to an extremely high level. In addition, the context of traditional network reciprocity [43] was further extended to the multilayer networks [13, 44, 45] (see [46] for a review).

Though the above mentioned achievements offer new insight into the issue of organization of collective behavior, the fundamental assumption that all individuals are homogenous and have the same properties seems inconsistent with realistic scenarios. While in the large-scale population evacuation, the population is typically composed of many small groups [47, 48]. Moreover, small groups are also ubiquitous in the vision-based investigation of pedestrian crowds [49]. Nevertheless, the behavioral evolution of small groups still remains largely unexplored. Currently, people mainly consider the behavioral evolution with two approaches. The first method based on the well-mixed population [6] aims to explain evolution of cooperative behavior by establishing the replicator equation with mean filed theory and evolutionary game theory [8–12]; while the second one (namely, agent-based method) [50] assumes the existence of a certain kind of spatial structure between the individuals and plans to study the behavioral evolution of the population [9, 23, 33, 36, 51]. In addition, the case of two heterogeneous groups has been explored [7, 24], but the interactions within and between small groups are partly considered. Furthermore, multiple heterogeneous small groups have been taken into consideration with the assumptions that the individuals belonging to different groups are well-mixed and all the interactions fall into the same dilemma [50]. Inspired by these achievements, an interesting question naturally poses itself, which we plan to address in what follows. If heterogeneity between small groups is incorporated into the behavioral evolution of evacuation scenarios, how do players choose their behavior?

In the present work, we consider the evolutionary games located in the population composed of two small groups. Within and between small groups, there exist different preferences among players. Then, we introduce characters of small groups in evacuation into simulation and aim to explore the regularity of behavioral evolution of small groups with heterogeneous preferences in population evacuation, by means of agent-based method in accordance with the game theoretical models built.

## Methods

Throughout this work, population is distributed on a *L×L* square lattice with periodic boundary conditions [52, 53]. Each player is initially designated as one of two behaviors: behavior 1 and behavior 2. Besides, since we consider the impact of small groups, the population is divided into small group 1 with probability *f* and small group 2 with the remaining probability 1-*f*, respectively. In particular, once two small groups are assigned, their ratios (*f* and 1-*f*) keep constant during the whole evolution process (namely, one player always belongs to the identical small group during the whole process).

With regard to evolutionary games, (since heterogeneity between small groups is taken into account,) we assume that there exist different dilemmas within and between small groups, which means that games are different within and between small groups. Along this line, there are three kinds of games, and the corresponding payoff matrices are $\left[\begin{array}{l} \left(r_{1},r_{1})(s_{1},t_{1}\right)\\ \left(t_{1},s_{1})(p_{1},p_{1}\right) \end{array}\right]$, $\left[\begin{array}{l} \left(R_{1},P_{2})(S_{1},S_{2}\right)\\ \left(T_{1},T_{2})(P_{1},R_{2}\right) \end{array}\right]$ and $\left[\begin{array}{l} \left(p_{2},p_{2})(t_{2},s_{2}\right)\\ \left(s_{2},t_{2})(r_{2},r_{2}\right) \end{array}\right]$, respectively. According to the principle of equivalent simplification of game theory [6, 9, 24], let B_*i*_ = *S*_*i*_–*P*_*i*_ B_*i+2*_ = *S*_*i*_–*P*_*i*_, *C*_*i*_ = *r*_*i*_–*t*_*i*_, *C*_*i+2*_ = *R*_*i*_–*T*_*i*_(*i* = 1,2) and then the payoff matrices can be translated into the form of Tables 1–3, respectively. In order to reflect heterogeneous preferences of different small groups, it is assumed that the *k*_th_ small group prefers behavior *k* (namely, players in small group 1 prefer behavior 1, and players in small group 2 are opt to behavior 2).

**Table 1:** The Payoff Matrix of the Interactions in Small Group 1(Simplified Form).

	Behavior 1	Behavior 2
**Behavior 1**	(*C*_1_,*C*_1_)	(*B*_1_,0)
**Behavior 2**	(0,*B*_1_)	(0,0)

**Table 2:** The Payoff Matrix of the Interactions between Small Groups 1 and 2(Simplified Form).

	Behavior 1	Behavior 2
**Behavior 1**	(*C*_3_,0)	(*B*_3_,*B*_4_)
**Behavior 2**	(0,0)	(0,*C*_4_)

**Table 3:** The Payoff Matrix of the Interactions in Small Group 2(Simplified Form).

	Behavior 1	Behavior 2
**Behavior 1**	(0,0)	(0,*B*_2_)
**Behavior 2**	(*B*_2_,0)	(*C*_2_,*C*_2_)

For each small group, there exists only one type of game, which but falls into different dilemmas based on different payoff ranking. Take Table 1 as example, the game will be prisoner’s dilemma game, harmony game, snowdrift game and stag hunt game if the payoffs satisfy *B*_1_<0,*C*_1_<0, *B*_1_>0,*C*_1_>0, *B*_1_>0,*C*_1_<0, and *B*_1_<0,*C*_1_>0 respectively. Accordingly, the equilibria of the four games are all defection, all cooperation, coexistence of defection and cooperation and all defection or all cooperation (relying on the initial condition), respectively.

When the game types are identical within small groups 1 and 2, such as two harmony games in Tables 1 and 3, some discussions can be provided as follows. If *C*_1_ = *B*_1_ = 0.1 and *C*_2_ = *B*_2_ = 10 the payoffs for Nash Equilibria of the two small groups are (0.1,0.1) and (10,10), respectively. Compared to small group 1, the players of small group 2 will suffer a great loss once deviating from the Nash Equilibrium. Therefore, the players of small group 2 are more reluctant to deviate from the strategy of Nash Equilibrium, which indicates that the payoff parameters of the games have significant influence on the cohesion of small groups, and then directly affect the behavioral evolution of the whole population. So are the other dilemmas. Hence, we define a key parameter: relative degree of preference for small group 1 (RDP for small group 1 in short), which depends on Nash Equilibria of games and will be represented by symbol *q* in this paper. If the Nash Equilibrium sets of Tables 1 to 3 are supposed to be $s_{\left(1\right)}^{*}$, $s_{\left(2\right)}^{*}$, $s_{\left(3\right)}^{*}$ respectively, then it can be defined as

$$q=\frac{\sum_{s_{\left(1\right)}^{*}\in s_{\left(1\right)}^{*}}u_{1}^{\left(1\right)}(s_{\left(1\right)}^{*})+\sum_{s_{\left(2\right)}^{*}\in s_{\left(2\right)}^{*}}u_{1}^{\left(2\right)}(s_{\left(2\right)}^{*})}{\sum_{s_{\left(3\right)}^{*}\in s_{\left(3\right)}^{*}}u_{2}^{\left(3\right)}(s_{\left(3\right)}^{*})+\sum_{s_{\left(2\right)}^{*}\in s_{\left(2\right)}^{*}}u_{2}^{\left(2\right)}(s_{\left(2\right)}^{*})}$$ (1)

Notably, the magnitude of *q* reflects the cohesion of small group 1 relative to small group 2. If *q*>1, it implies that the cohesion of small group 1 is greater than that of small group 2; otherwise the better is for small group 2.

After the definition of payoff details, the game is iterated forward in accordance with the sequential simulation procedure comprising the following elementary steps. First, each player gets its payoff by playing the games with its four immediate neighbors, irrespective of same or different small groups. Then, every player will update its strategy by adopting the strategy of neighbor who possesses the highest total payoff in the present round. We use the synchronous updating so that each player updates its strategy simultaneously.

## Results

In this section, we first set the parameters of the game models according to the scenarios of crowd evacuation, and then simulate behavioral evolution of small groups by varying two key parameters: *f* and *q*.

The individuals in the same small group have the identical preference, but some players sometimes may deviate from the preferred behavior. This character is in line with harmony game, so the dilemma within the same small group is featured as harmony game. Namely, game types of Tables 1 and 3 are harmony game, and accordingly the parameters of the games satisfy C_*i*_,*Bi*>0(*i* = 1,2). In the process of emergency evacuation, there also exists the interaction between different small groups, although whose preference is different. The aim of each player in the interaction is very clear, which resembles the scenario of snowdrift game. Thus it is assumed that the interaction between two small groups mimics the snowdrift game, that is, the payoff parameters of Table 2 satisfy *C*_*i*_>0,*B*_*i*_<0(*i* = 3,4). Since the game parameters are confined to certain ranges stated above, it can be derived straightforwardly that

$$q=\frac{C_{3}+C_{1}}{C_{4}+C_{2}}$$ (2)

according to Equation (1).

Results of computer simulations presented below are obtained on populations comprising *L* = 100 (We have verified our results for different sizes *L* = 200,400, and the main results are robust to the size *L*). The results of system is averaged on 500 steps after the fluctuation is smaller than 0.01 with 500 continuous steps. Moreover, since the heterogeneous preferences may introduce additional disturbances, final results are averaged over up to 10 independent runs for each set of parameter values in order to assure suitable accuracy.

### The Impact of Fraction of Small Group 1 on Evolution

**Fig 1:**
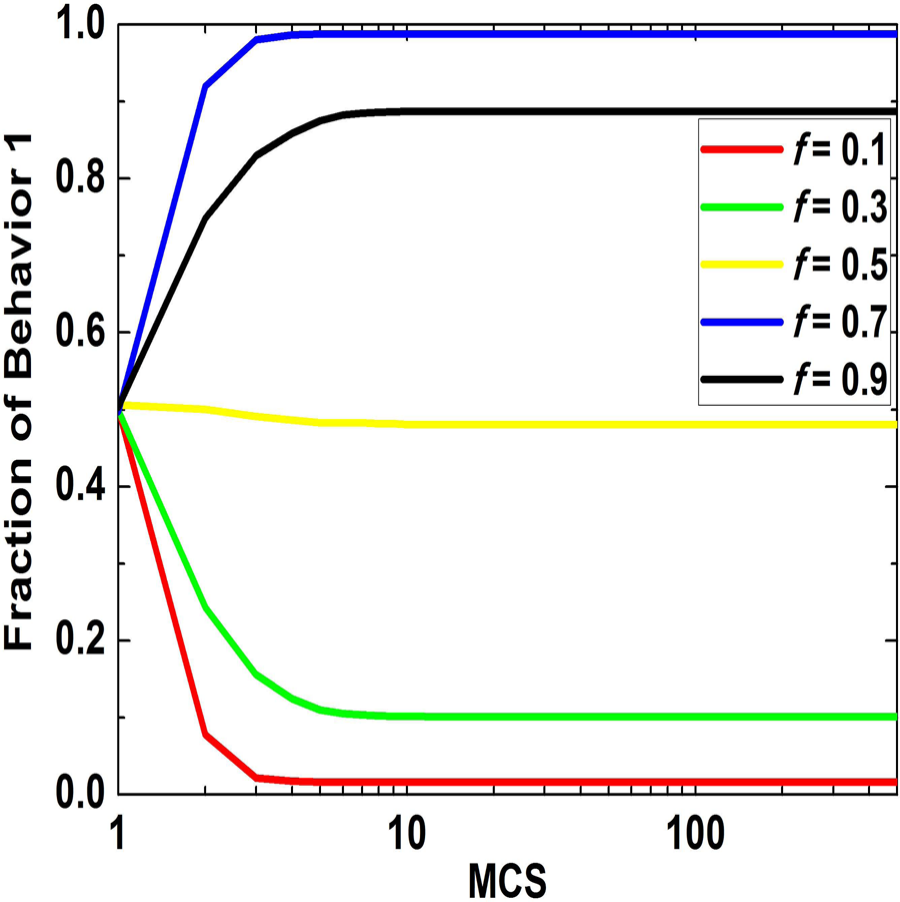
Fraction of behavior 1 in population which changes over time when the game parameters are constant. In the figure, the five curves correspond to five different fractions of small group 1, namely 0.1, 0.3, 0.5, 0.7, 0.9. The used payoff parameters are [*B*_1_, *B*_2_, *B*_3_, *B*_4_, *C*_1_, *C*_2_, *C*_3_, *C*_4_] = [2, 2, –1, –1, 1, 1, 2, 2].

At first, the impact of the fraction of small group 1, namely *f*, on behavioral evolution of population is explored with fixed $q=\frac{C_{3}+C_{1}}{C_{4}+C_{2}}=1$. Fig. 1 shows how the ratio of behavior 1 in population changes over time for different values of *f*. As there is no randomness, the collective behavior reaches the stabile state at even about the 50th step. It is clear that with the increment of *f*, proportion of behavior 1 in population monotonously increases. For a small value, the system fast declines to disappearance of behavior 1. However, when *f* is sufficiently large, behavior 1 is will completely dominate the system.

**Fig 2:**
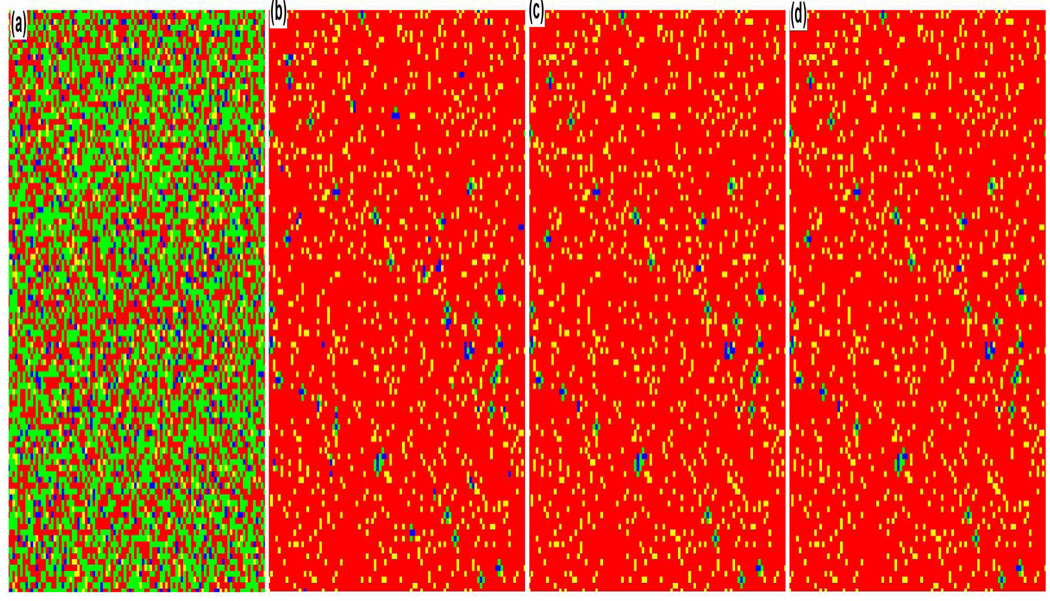
Direct observations of behavioral evolution for the whole population when *f* = 0.1 and *q* = 1. Snapshots are taken at *t* = 1, 3, 100 and 500 MC steps. In the figure blue, yellow, green and red are used to represent behavior 1 in group 1, behavior 2 in group 1, behavior 1 in group 2 and behavior 2 in group 2 respectively. The game parameters in the numerical simulation are set as [*B*_1_, *B*_2_, *B*_3_, *B*_4_, *C*_1_, *C*_2_, *C*_3_, *C*_4_] = [2, 2, –1, –1, 1, 1, 2, 2].

In order to provide a visual validation of the observations, we show the snapshots of the behavioral evolution at *t* = 1, 3, 100 and 500 MC steps (see Fig. 2), in which *f* and *q* are 0.1 and 1, respectively. At the beginning of the evolution, small group 2 accounts for the great majority of the whole population, while fractions of behavior 1 in both small groups are equal to 0.5. As time evolves, when the evolution is stable, in both small groups the individuals choosing behavior 2 dominate and the remaining ones in minority who adopt behavior 1 are aggregating in clusters. Therefore, it can be concluded that as the fraction of small group 2 is greater than that of small group 1, the behavior which small group 2 prefers is more easily spread in the population and becomes the normative behavior of the population ultimately.

**Fig 3:**
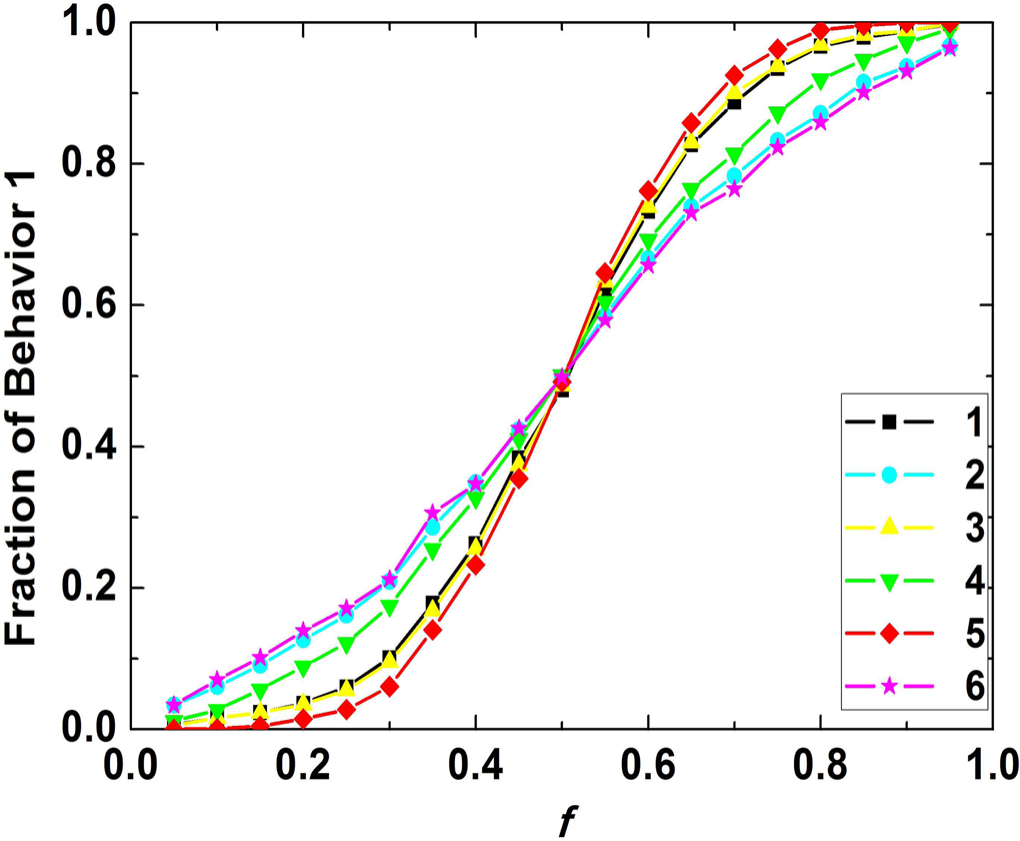
Relationship between the fraction of behavior 1 in population and the fraction of small group 1. The curves in the figure are with the same *q* (equal to 1), but the game parameters are not the same. For parameter group 1, [*B*_1_, *B*_2_, *B*_3_, *B*_4_, *C*_1_, *C*_2_, *C*_3_, *C*_4_] = [2, 2, –1, –1, 1, 1, 2, 2]. For parameter group 2, [*B*_1_, *B*_2_, *B*_3_, *B*_4_, *C*_1_, *C*_2_, *C*_3_, *C*_4_] = [1, 1, –1, –1, 1, 1, 2, 2]. For parameter group 3, [*B*_1_, *B*_2_, *B*_3_, *B*_4_, *C*_1_, *C*_2_, *C*_3_, *C*_4_] = [2, 2, –2, –2, 1, 1, 2, 2]. For parameter group 4, [*B*_1_, *B*_2_, *B*_3_, *B*_4_, *C*_1_, *C*_2_, *C*_3_, *C*_4_] = [1, 1, –2, –2, 1, 1, 2, 2]. For parameter group 5, [*B*_1_, *B*_2_, *B*_3_, *B*_4_, *C*_1_, *C*_2_, *C*_3_, *C*_4_] = [2, 2, –1, –1, 2, 2, 2, 2]. For parameter group 6, [*B*_1_, *B*_2_, *B*_3_, *B*_4_, *C*_1_, *C*_2_, *C*_3_, *C*_4_] = [2, 2, –1, –1, 1, 1, 3, 3].

In order to study regularity of the impact of fraction of small group 1 on the fraction of behavior 1 in the population and discuss the robustness of this regularity, we set *q* equal to 1 and choose 6 groups of game parameters, and the results of the simulations are depicted in Fig. 3. The six curves illustrate that with *f* increasing, the fraction of behavior 1 in the population after behavioral evolution will also increase. In detail, if *f* is smaller than 0.25, fraction of behavior 1 in the population will be smaller than 0.2, so in this case the majority of the population show the normative behavior preferred by small group 2 ultimately. Likewise, it is recognized that the population prevailingly exhibit the behavior preferred by small group 1 if *f* is larger than 0.75. The regularity is similar for different game parameters, so the relationship between fraction of behavior 1 in population and *f* is robust for different game parameters (*q* is supposed to be constant).

To sum up, fraction of small group 1 has an important impact on the behavioral evolution of the population and the impacting curve is monotonically increasing. When *q* is close to 1, it is easier for the larger small group to establish the behavior it prefers as the normative behavior. In addition, this regularity is robust for different parameters.

### The Impact of RDP for Small Group 1 on Evolution

**Fig 4:**
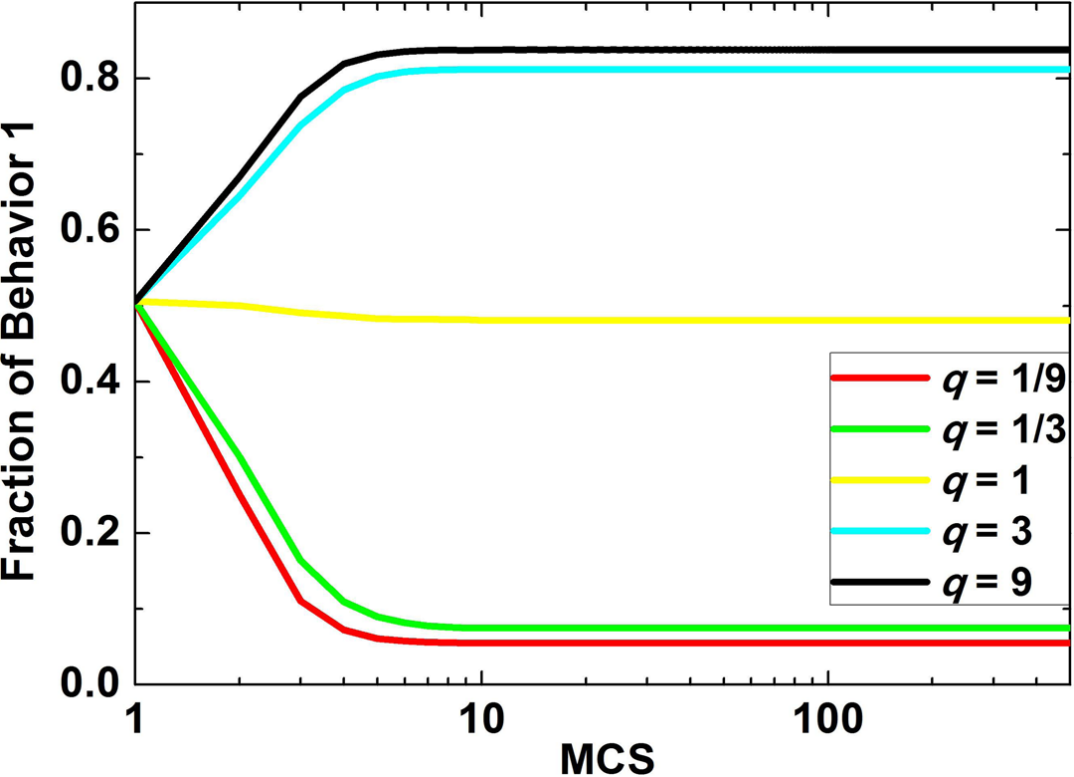
Fraction of behavior 1 in population which changes over time, when the fraction of small group 1 is fixed. In this figure, the five curves correspond to five different values of *q*. In detail, in the simulations *f* = 0.5, and the game parameters are selected as [*B*_1_, *B*_2_, *B*_3_, *B*_4_, *C*_1_, *C*_3_] = [2, 2, –1, –1, 1, 2], *C*_2_ = *C*_1_/*q*, *C*_4_ = *C*_3_/*q* and values of *q* are chosen as 1/9, 1/3, 1, 3, 9, respectively.

Next, we inspect how RDP for small group 1 impacts behavioral evolution when fraction of small group 1 is fixed. Fig. 4 shows time courses of behavior 1 in the population corresponding to different values of *q*. Similar to Fig. 1, the curves will reach the stable state at about the 50th step. Importantly, as *q*increases, the fraction of behavior 1 in population will also increase.

**Fig 5:**
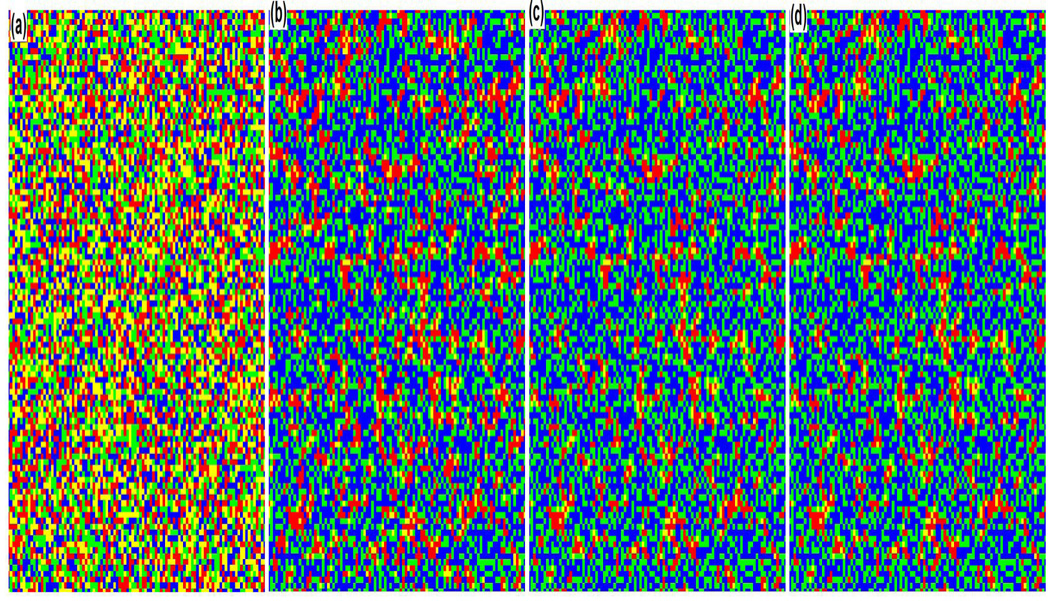
Direct observations of behavioral evolution for the whole population when the values of *f* and *q* are 0.5 and 9 respectively. Snapshots are taken at *t* = 1, 3, 100 and 500 MC steps. The meanings of the four colors are the same with Fig. 2. Specifically, the payoff values are set as [*B*_1_, *B*_2_, *B*_3_, *B*_4_, *C*_1_, *C*_2_, *C*_3_, *C*_4_] = [2, 2, -1, -1, 1, 1/9, 2, 2/9] in the numerical simulation.

In order to feature the observations in more details, we have taken snapshots of the evolution at *t* = 1, 3, 100 and 500 MC steps (see Fig. 5), in which *f* and *q* are set as 0.5 and 9 respectively. At the beginning of the evolution, the four types of agents are equally distributed on the lattice. As time evolves, when the behavioral evolution of the two small groups is stable, the individuals with behavior 1 account for the great majority in both small groups, although there are a few agents adopting behavior 2 and they are gathering in clusters. Therefore, when fractions of two small groups are close and RDP for small group 1 is larger than 1, it can be concluded that small group 1 will win in establishing the behavioral norm due to stronger cohesion although there is no advantage in scale for small group 1. In other words, the behavior small group 1 prefers is more easily spread in the population and will become the normative behavior of the population ultimately.

**Fig 6:**
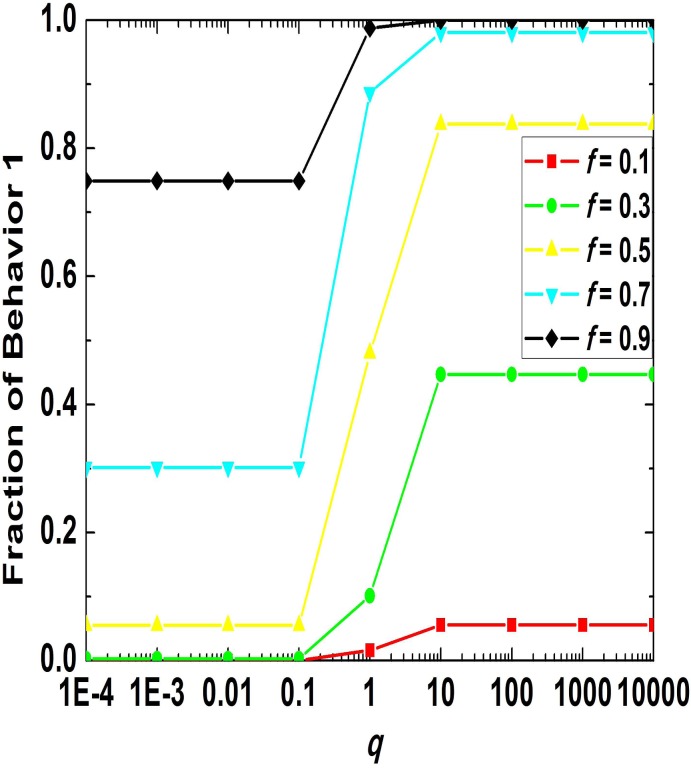
Relationship between the fraction of behavior 1 in population and RDP for small group 1. In the numerical simulations corresponding to the figure, the payoff parameters adopted are specified as [*B*_1_, *B*_2_, *B*_3_, *B*_4_, *C*_1_, *C*_3_] = [2, 2, –1, –1, 1, 2], *C*_2_ = *C*_1_/*q*, *C*_4_ = *C*_3_/*q*.

Aiming to investigate the impact of *q*on behavioral evolution and discuss the law of evolution for different values of *f* we have scanned a wide range of parameters and the simulation results are depicted in Fig. 6. As can be seen, when *f* is fixed, the fraction of behavior 1 in the population after behavioral evolution comes to an end is positively correlative to *q*, and *q* has the greatest influence on the result of evolution when it lies between 0.1 and 10. Beyond this scope, in particular when *q*is smaller than 0.01 or larger than 100, its impact can be neglected and behavioral evolution of population in this case mainly depends on *f*.

To sum up, RDP for small group 1 has an important impact on the behavioral evolution of the population. It imposes its influence by the relative cohesion of small group 1. Through the study on the impact of *q*on behavioral evolution for small groups 1 and 2 when *f* is selected from a series of fixed values, it can be claimed that when *q*is in a center range, its influence is very remarkable, whereas when it is out of the range, the behavioral evolution of the population mainly relies on *f*.

**Fig 7:**
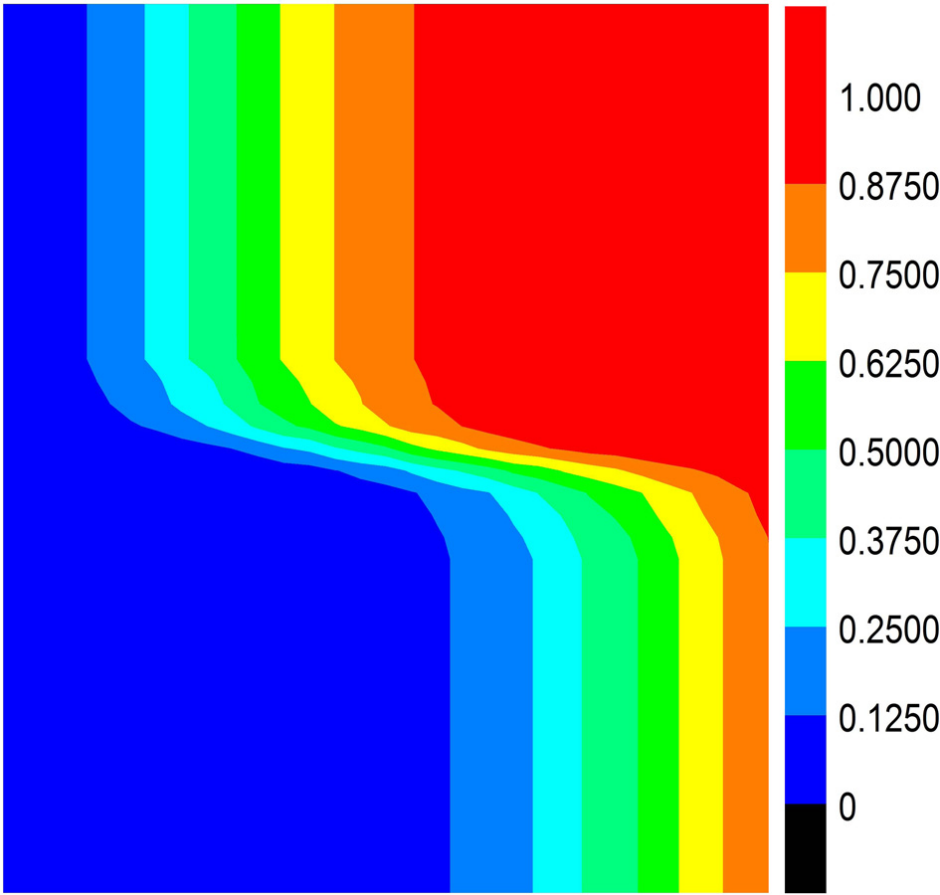
Relationship between the fraction of behavior 1 in the population and two variables, namely, *f* and *q*. The game parameters used in this figure are the same with those in Fig. 6.

Finally, in order to understand the relationship between the result of behavioral evolution and *f* as well as *q*, we have carefully skimmed a wide range of values for them. The results are demonstrated in Fig. 7. It can be seen from Fig. 7 that the stratification phenomenon is very clear, so it can be concluded that the two quantities have important impacts on the behavioral evolution of the population. When *f* and *q* are relatively large, the behavior small group 1 prefers will become the normative behavior of the population; likewise, when the two values are small, small group 2 will establish the behavioral norm.

## Conclusions

To conclude, the evolutionary games in small groups with heterogeneous preferences have been considered. Within and between small groups, there will be different dilemmas. Based on a great number of computation simulations, it can be concluded that the final behavior of the whole population is closely related with the ratio of players in one certain small group directly. Moreover, it is shown that heterogeneous degree of preference for different small groups also directly determines the behavior traits of the system, which may shed new light into the realistic social observations. We hope that our proposed framework can bring more effective solutions to social dilemmas.

## Supporting Information

S1 Dataset The dataset of Fig. 1. (XLSX)

S2 Dataset The dataset of Fig. 2. (XLSX)

S3 Dataset The dataset of Fig. 3. (XLSX)

S4 Dataset The dataset of Fig. 4. (XLSX)

S5 Dataset The dataset of Fig. 5. (XLSX)

S6 Dataset The dataset of Fig. 6. (XLSX)

S7 Dataset The dataset of Fig. 7. (XLSX)
